# High Temperature Oxidation Behavior of an Equimolar Cr-Mn-Fe-Co High-Entropy Alloy

**DOI:** 10.3390/ma14154259

**Published:** 2021-07-30

**Authors:** Lin Wang, Quanqing Zeng, Zhibao Xie, Yun Zhang, Haitao Gao

**Affiliations:** 1 College of Mechanical and Electrical Engineering, Central South University, Changsha 410083, China; lin_wang@csu.edu.cn (L.W.); xiezhibao@csu.cn (Z.X.); 2 State Key Laboratory of High Performance Complex Manufacturing, Central South University, Changsha 410083, China; 3 Light Alloys Research Institute, Central South University, Changsha 410083, China; 173811017@csu.edu.cn

**Keywords:** metals and alloys, 3D printing, oxidation

## Abstract

The oxidation behavior of an equimolar Cr-Mn-Fe-Co high-entropy alloy (HEA) processed by 3D laser printing was investigated at 700 °C and 900 °C. The oxidation kinetics of the alloy followed the parabolic rate law, and the oxidation rate constant increased with the rising of the temperature. Inward diffusion of oxygen and outward diffusion of cations took place during the high-temperature oxidation process. A spinel-type oxide was formed on the surface, and the thickness of the oxide layer increased with the rising of experimental temperature or time. The exfoliation of the oxide layer took place when the test was operated at 900 °C over 12 h. During oxidation tests, the matrix was propped open by oxides and was segmented into small pieces. The formation of loose structures had great effects on the high-temperature oxidation resistance of the HEA.

## 1. Introduction

After originally proposed by Yeh et al. [1], high-entropy alloys (HEA) have attracted increasing attention due to their excellent mechanical properties [2,3], and corrosion resistance [4,5], etc. Gludovatz et al. [6] reported a deformation mechanism for Cr-Mn-Fe-Co-Ni HEA transiting from planar-slip dislocation activity to nanotwinning as the temperature decreased from room temperature to cryogenic temperature, with cryogenic temperature offering better mechanical properties. Otto et al. [7] systemically investigated the relationship between temperatures (between −196 and 800 °C), microstructures and the tensile properties of a Cr-Mn-Fe-Co-Ni HEA. The alloy showed a significant improvement in yield strength, fracture elongation and ultimate tensile strength with the decreasing temperature. What’s more, some deformation-induced twins were observed as the tensile test was interrupted after more than 20% strain when the test temperature was lower from 20 °C to −196 °C. Yong et al. [8] proposed that the separation of nanoscale phase could significantly promote the yield strength of Mn-Fe-Co-Ni-Cu alloy. Keil et al. [9] systematically researched the equiatomic composition for mechanical properties optimum of (Cr-Mn-Fe-Co)x-Ni1-x and quantitatively investigated the saturation grain size, hardness and strain rate sensitivity with the variation of Ni element composition.

High-temperature resistant materials have been widely used in the nuclear industry, civil industry, military industry, etc. However, there were little researches focused on the oxidation behavior of HEA. Kai et al. [10] investigated the oxidation behavior of an equimolar Cr-Mn-Fe-Co-Ni HEA at 950 °C in various oxygen-containing atmospheres with PO_2_ = 10, 1.0 × 10^3^, 2.1 × 10^4^ and 1.0 × 10^5^ Pa, respectively. They found that the oxidation kinetics of the alloy followed a single-stage parabolic rate curve with raising oxygen pressure. While the generated triplex scales consisted of an exclusive Mn_3_O_4_ outer layer, a hetero phasic intermediate layer of Mn_3_O_4_, (Mn, Cr)_3_O_4_, and Cr_2_O_3_, and an exclusive Cr_2_O_3_ inner layer. Gorr et al. [11] studied the microstructure and high-temperature oxidation behavior of Mo-W-Al-Cr-Ti HEA which was processed by casting and heat treatment. The results revealed that the mass gain obeyed a parabolic rate law when this alloy was exposed to air at 1000 °C for 40 h, indicating that the oxide scale grew by solid-state diffusion. Pouraliakbar et al. [12] attempted to produce novel high-temperature materials by increasing the content of AlTiZr and CuFeMo in the multicomponent alloying system (CoCrNi)_1−x−y_(AlTiZr)_x_(CuFeMo)_y_. Yong et al. [13] researched the high-temperature stability of Cu-rich filaments and discovered that the dual fcc phase alloy (e.g., Cr-Fe-Co-Cu-Ni and Cr-Fe-Co-Cu1.71-Ni HEA) owed excellent strengths even after annealing at 1000 °C. This was owing to the low internal strain energy which was resulted from continuous recrystallization during deformation processing. As mentioned in the previous literature, there are numerous high-temperature oxidation resistance applications between 700 °C to 900 °C. Thus a thorough grasp of the high-temperature oxidation in the temperature range is necessary.

HEAs have potential applications at high temperatures ranging from 700 °C to 900 °C. In this study, the high-temperature oxidation mechanism of a Cr-Mn-Fe-Co HEA produced by 3D laser printing was investigated.

## 2. Materials and Methods

Initially, Cr, Mn, Fe and Co metal powders were fully mixed in equal moles. In addition, the equimolar Cr-Mn-Fe-Co HEA with a size of 20 × 60 × 12 mm^3^ was produced by a numerical control system (3D-ZM, Dalian, China) with a laser scanning speed of 600 mm/min and laser power of 1000 W. Six cubes with the size of 6 × 6 × 6 mm^3^ were cut from the initial alloy utilizing wire electrical discharge machine. Each surface was polished on SiC papers up to 1600 grit, cleaned by alcohol in an ultrasonic machine and dried in high pressure air.

The oxidation experiment was operated in a non-vacuum heating furnace. It was heated in the synthetic air (79% N_2_ and 21% O_2_) to the temperatures of 700 °C and 900 °C up to 48 h. After 48 h, the specimens were cooled down to room temperature in the furnace under laboratory air. Analytical balance was used to accurately measure the weight of samples. An X-ray diffractometer (D8 ADVANCE, BRUKER, Leipzig, Germany) equipped with Cu kα (λ = 1.5406 Å), scan step size of 0.02 deg. and a scan rate of 1 deg.min-1 was utilized to identify phases for the physic-chemical characterization. A scanning electron microscopy (SEM) equipped with an energy dispersive spectrometer (EDS) on the platform of MIRA 3 LMU (TESCAN, Brno, The Czech Republic) was used to identify the images and the element distribution. In the EDS experiments, the accelerating voltage was 15 kV, and the distance between two measuring points was 0.35 μm.

## 3. Results and Discussion

### 3.1. Oxidation Properties of Cr-Mn-Fe-Co HEA

**Figure 1:**
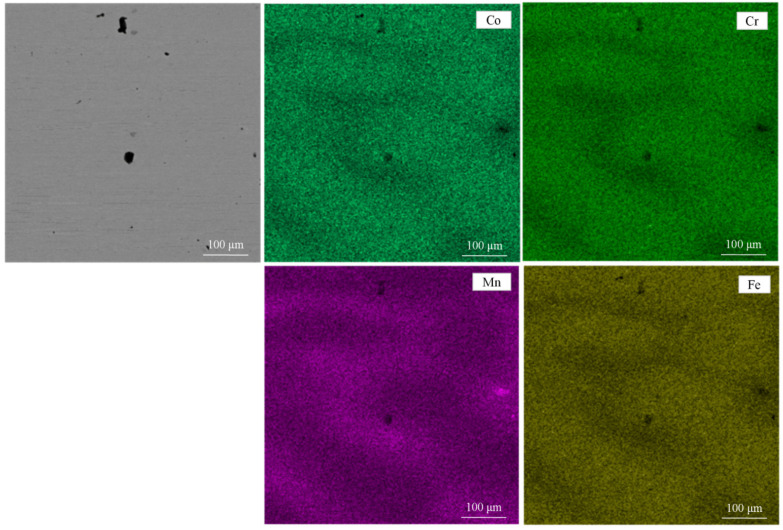
EDS results of the initial HEA.

Figure 1 shows the EDS results of the initial composition of the alloy. Each element is evenly distributed in matrix and the content of Co, Cr, Fe, and Mn were 24.67%, 24.25%, 25.72%, and 25.37%, respectively, which has been shown in Table 1.

**Figure 2:**
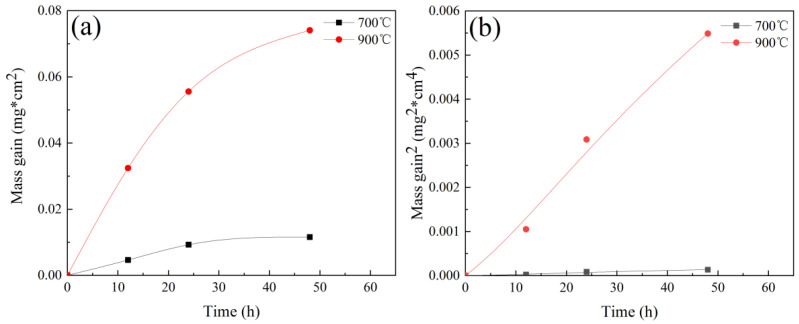
The mass gain of HEA: (**a**) Weight gain versus oxidation time curves; (**b**) Parabolic plot.

The mass gain data were obtained from separated samples for each data point. Figure 2 shows the mass gain of the HEA at 700 °C and 900 °C over a period of 48 h. The mass gain (If there is oxide exfoliation, it was took into account.) increased at a given oxidation period as the oxidizing temperature raised. Furthermore, the mass gain rate of the alloy at a certain temperature was gradually decreased with the increasing of oxidation time. Mass gain after 48 h oxidation tests was 0.012 and 0.074 g/cm^2^ for 700 °C and 900 °C, respectively. The oxidation kinetics of the alloy followed the parabolic rate law at both two temperatures as illustrated in oxidation curves (Figure 2a). The parabolic rate constant $K_{p}$ can be obtained by $K_{p}=\frac{{\left(\Delta m\right)}^{2}}{A^{2}\times t}$, where, Δm represents mass gain, A represents sample area, t represents oxidation time. The parabolic rate constant $K_{p}$ of 2.79 mg^2^/cm^4^/h at 700 °C sharply increased to 114 mg^2^/cm^4^/h at 900 °C after 48 h. Compared to the oxidation behavior of equimolar Cr-Mn-Fe-Co-Ni HEA [14], its parabolic rate constant of 0.13 mg^2^/cm^4^/h at 900 °C verified the significant effect of Ni element, on the sluggish diffusion of alloy’s high-temperature oxidation resistance. Chromium, acting as a getter for oxygen in the alloy during the initial stage, can lower the oxygen solubility in the alloy and reduce the internal oxidation speed of other elements [15]. With relative high content of chromium element, the relative low rate constants were obtained [16]. However, the rate constant increased rapidly compared to the Cr-Mn-Fe-Co-Ni alloy through the increase in the chromium element. Firstly, the oxides of Mn presented in the areas where cracks appeared and the relative high content of Mn may result in cracked oxides. In addition, Ni element can significantly increase the oxidation resistance at high temperature through reducing the volatility of Cr_2_O_3_ by forming a solid solution in the oxides [14]. The relative discussion has been added in the paper.

**Figure 3:**
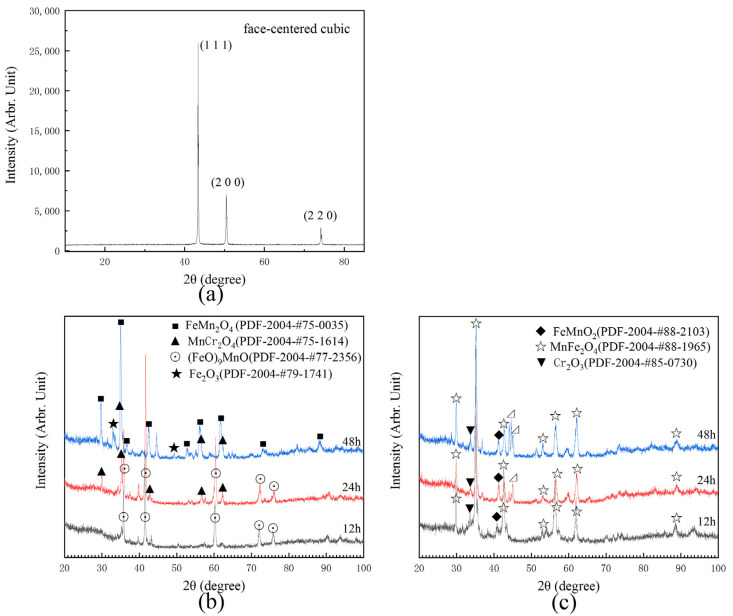
XRD results of HEA: (**a**) XRD spectra of the initial alloy; (**b**,**c**) XRD spectra after isothermal oxidation tests at 700 °C and 900 °C, respectively.

Figure 3a shows the XRD results of initial Cr-Mn-Fe-Co HEA, which is a single FCC solid solution phase [17]. Figure 3b,c present XRD analysis of Cr-Mn-Fe-Co HEA after isothermal oxidation experiments at 700 °C and 900 °C. After oxidizing at 900 °C over 12 h, the oxide layer on the surface was exfoliated. Apart from FeO and MnO identified on the surface oxidized at 700 °C for 12 h, some MnCr_2_O_4_ was also detected when the alloy was oxidized at 700 °C for 24 h, while Fe_2_O_3_ and FeMn_2_O_4_ appeared when the oxidation duration was prolonged to 48 h. Furthermore, MnFe_2_O_4_, MnFeO_2_ and Cr_2_O_3_ were observed on the surface when the specimens were oxidized at 900 °C. The thickness of oxide layers increased with the increase in the oxidation time, indicated by the increase in the peak intensity.

### 3.2. High-Temperature Surface Oxidation Mechanism of Cr-Mn-Fe-Co HEA

**Figure 4:**
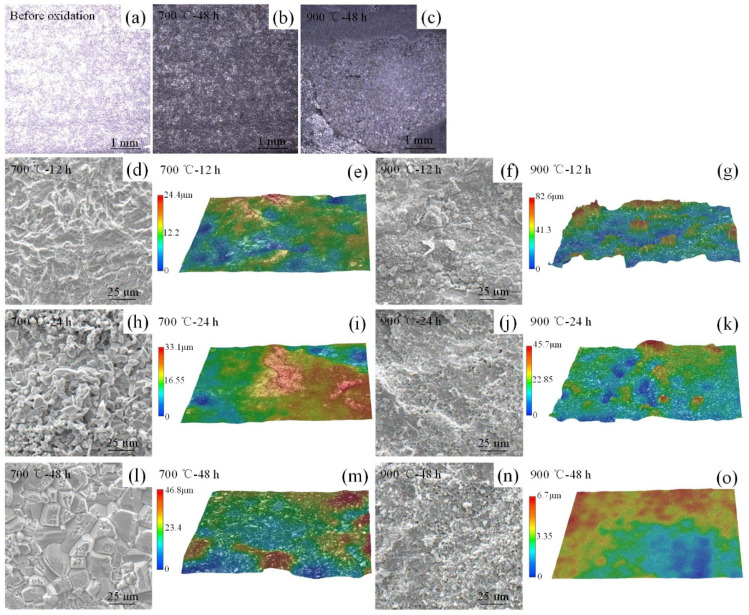
Surfaces micrographs of Cr-Mn-Fe-Co HEA at different temperatures and various times.

Figure 4 shows the surfaces micrographs of the HEA before and after the high-temperature oxidation test. Compared to the initial surface and the surfaces oxidized at 700 °C for 48 h, the surface after oxidation at 900 °C showed clear cracks, holes and obvious spallation of oxide layer (Figure 4a–c). The oxidation on a certain surface was obviously different on different regions, which resulted from the different speeds of oxidation penetration through grains and grain boundaries and the element diffusion. The spinel shape oxides were observed on all surfaces after oxidation tests, which was similar to the high-temperature oxidation corrosion behavior of Cr-Mn-Fe-Co-Ni HEA preferring grain boundary area [14]. Compared to the magnified images of the specimens after oxidation at 700 °C in Figure 4d,e,h,l,m, the surface after oxidized at 900 °C (Figure 4f,g,j,k,n,o) presented numerous pores and cracks and the oxidation layer peeled off. After the peeling, the surface of the specimen oxidized at 900 °C for 48 h represented numerous pores and cracks again. As a result, the maximum roughness increased from 24.4 μm to 46.8 μm with the test time increasing from 12 h to 48 h, while it was reasonable for a curtained trend of the maximum roughness measured at 900 °C.

**Figure 5:**
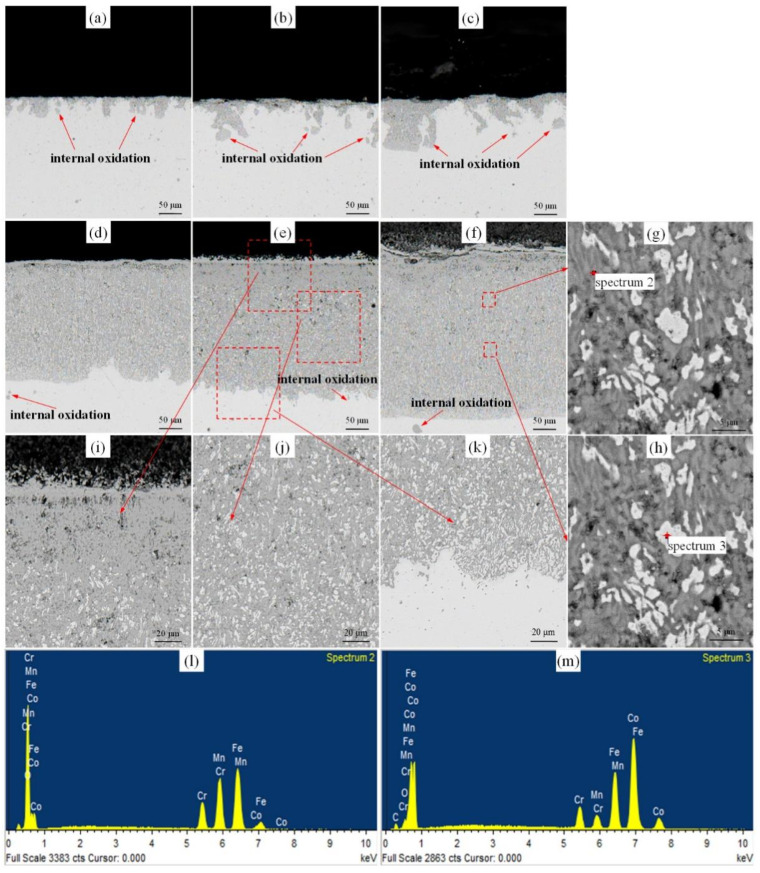
Cross-section images of Cr-Mn-Fe-Co HEA after oxidation test at 700 °C for (**a**) 12 h, (**b**) 24 h, (**c**) 48 h, and at 900 °C for (**d**) 12 h, (**e**) 24 h, (**f**) 48 h. (**g**,**h**) for one magnification image of (**f**), (**i**–**k**) three magnified images of (**e**), (**l**) the element distribution of spectrum 2, (**m**) the element distribution of spectrum 3.

Figure 5 shows the vertical cross-sections images of the oxides. The oxide thickness increased with the oxidation time and temperatures. When tested at 700 °C, some oxides and voids were observed on the surface of HEA as well as internal oxidation. Numerous cracks were formed when the oxidation time extended to 24 h. Dense oxides formed when the tests were operated at 900 °C and spallation of the oxide scales was observed, caused by interaction between different oxides, defects and matrix. The details of specimens after oxidation at 900 °C for 24 h were magnified in Figure 5i–k. The middle and bottom parts of the oxide layer consisted of oxides (FeO and MnO) and metal matrix simultaneously. The upper part of oxidation layer (MnFe_2_O_4_, MnFeO_2_ and Cr_2_O_3_) was composed of oxides and a large number of holes, which may result in easier diffusion of oxygen to the matrix and reduce the resistance of oxidation corrosion at high temperature. Furthermore, the chemical composition of the oxide in Figure 5f was analyzed and the results were shown in Figure 5l,m. An oxygen content of 57.74% (atomic) was detected at spectrum 2 (Figure 5l), verified to be oxides. While it had a small amount of oxygen element (atomic, 5.69%) at spectrum 4 (Figure 5m). The matrix was propped open by oxides and segmented into small pieces as shown in Figure 5i–k, resulting in materials failure.

**Figure 6:**
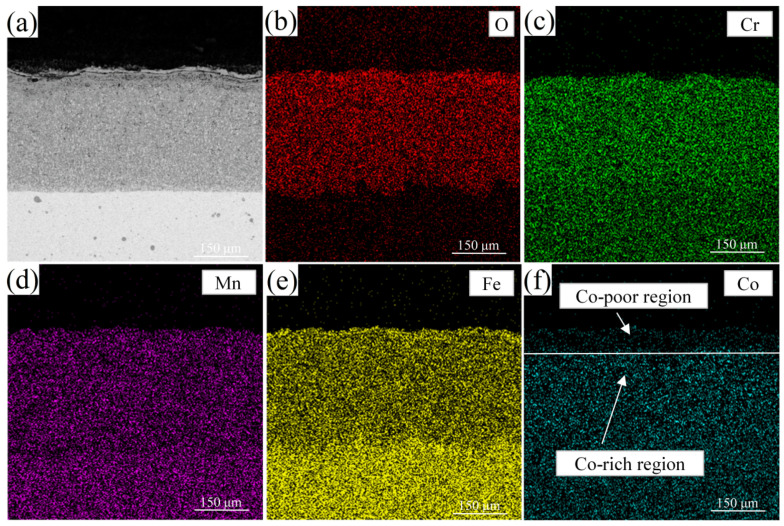
EDS results of Cr-Mn-Fe-Co HEA after isothermal oxidation tests at 900 °C for 48 h.

The element distribution after oxidation is shown in Figure 6. Some voids were observed, which were Kirkendall pores, resulting from different diffusion of Cr, Mn, Fe and Co. An unstable Cr-rich, Mn-rich and Fe-rich oxide layer, respectively inferred to (Mn, Cr)_3_O_4_, Cr_2_Mn_3_O_8_ and (Mn, Fe)_2_O_4_ formed during the test. They were easy to fall off from the matrix after oxidation at 900 °C. Cr, Mn and Fe had higher diffusion rates than Co, resulting in the development of Co-poor region and Co-rich region, as shown in Figure 6f. Fe and Co produced an oxidation layer in MnFe_2_O_4_, MnFeO_2_ and Cr_2_O_3_ at high temperature, leading to a reduction in the oxidation resistance of Cr-Mn-Fe-Co HEA. After oxidation at 900 °C, few oxides pegs were observed resulting in a weakened connection between oxides and the substrate.

The instantaneous rate constant $K_{p}$ decreased with the incremental oxidation time. Activation energy ($E_{a}$) was calculated from the $K_{p}$ values and the corresponding test temperatures. It gave the value of $E_{a}=212\mathrm{kJ}/\mathrm{mol}$. The value ranged between 250 and 290 kJ/mol of chromia (Cr_2_O_3_) formation alloys during the oxidation as previously reported in the literature [18,19]. It was in good agreement with the observation in Figure 4 and Figure 5. A chromatic scale, a diffusion barrier, appeared in the scale. However, compared to the Cr-Mn-Fe-Co-Ni HEA, the absence of Ni element greatly reduced the hindrance of cation diffusion and led to a good oxidation behavior.

## 4. Conclusions

The oxidation behavior of Cr-Mn-Fe-Co HEA produced by 3D laser printing was investigated at 700 °C and 900 °C up to 48 h. The main conclusions are as follows:

(1) The initial alloy was verified as FCC random solid solution. The mass gain in the oxidation process for 48 h was measured to be 0.02 and 0.15 g/cm^2^ at 700 °C and 900 °C, respectively. 

(2) MnFe_2_O_4_, MnFeO_2_ and Cr_2_O_3_ were detected by the XRD analysis on the specimen surface. Some surface cracks were observed, especially for those oxidized at 900 °C. The spallation of oxide layer took place when the alloy was tested at 900 °C. The high-temperature oxidation resistance of the alloy sharply decreased when the test temperature increased from 700 °C to 900 °C, leading to the wide application under 700 °C.

## Figures and Tables

**Table 1:** Chemical composition of the HEA.

Composition (wt.%)	Co	Cr	Fe	Mn
The initial HEA	24.67	24.25	25.72	25.37

## Data Availability

The data presented in this study are available on request from the corresponding author.
